# Supplementary material for: Chromothripsis during telomere crisis is independent of NHEJ, and consistent with a replicative origin
Source: Genome Res. 2019 May;29(5):737–49. doi: 10.1101/gr.240705.118 (PMC6499312; doi:10.1101/gr.240705.118)
Supplement: Supplemental Material [file supp_gr.240705.118_Supplemental_file_1.zip › contigs/annotated_contigs/DB79/contig.2.DB79_length_264_mean_cov_3.97727272727.docx]

**DB79_length_264_mean_cov_3.97727272727**

```
TATTTTGAATTATTTATTTCACAGAAGAGACGAGGAAAGTAGGGAGAGGAAGGAAACAAAGAGGAGG|C|ATCTCTTTCTGTGTCCACA
 >chr6:125543689-125543757 - E=1e-29                                >chr7:151420150-151
GCCCCGGCTCTGAGAGGTGGACCACAGTGACGTTGTGCATCTCCTGGGCTGAATGTCAGTTTAGAATCAGTGTTCTGTCACCTCCAATA
420347 + E=1e-107
CCCTTTCCCTAATGCCCAGTTGCCCTGGTAAAAGCCATAAGTCCCTTTGGGGAAGAACGGGAGAGAGGTTAATAGTATACATTTTTGG
```
